# Does the ‘Educational Alliance’ conceptualize the student - supervisor relationship when conducting a master thesis in medicine? An interview study

**DOI:** 10.1186/s12909-023-04593-7

**Published:** 2023-08-28

**Authors:** Michael Brenner, Anja Nikola Weiss-Breckwoldt, Flurin Condrau, Jan Breckwoldt

**Affiliations:** 1 Department of Internal Medicine, Swiss Paraplegic Group, Nottwil, Switzerland; 2 Psychiatric Outpatient Clinic, Ambulatorium am Roemerhof, Zurich, Switzerland; 3 Center for Medical Humanities, Institute for Biomedical Ethics and History, University of Zurich, Zurich, Switzerland; 4 Institute of Anesthesiology, University Hospital Zurich, Raemistr. 100, Zurich, CH-8091 Switzerland

**Keywords:** Educational alliance, Feedback, Master thesis, Scientific training, Science curriculum, Supervision, Undergraduate education

## Abstract

**Background:**

Completing a master thesis (MT) is mandatory in many undergraduate curricula in medicine but a specific educational framework to guide the supervisor-student relationship during the MT has not been published. This could be helpful to facilitate the MT process and to more effectively reach the learning objectives related to science education in medicine. An attractive model for this purpose is the ‘Educational Alliance’ (EA), which focusses on the three components ‘*clarity and agreement on (a) goals, (b) tasks and (c) relationship & roles*’. This study investigated factors that can either facilitate or hinder the process of MTs, and related these to the components of the EA.

**Methods:**

We conducted semi-structured face-to-face interviews with 20 students and – separately – with their 20 corresponding supervisors, after the MT had been accepted. The interviews included open questions on factors facilitating or hindering the success of the MT. Audio recordings of the interviews were anonymized and transcribed, and then analysed by qualitative content analysis. Also, quantitative data were gathered on satisfaction with the MT process and the supervisory quality (using Likert-type questions).

**Results:**

We were able to analyse all 40 interviews, related to 20 MTs. From the transcripts, we extracted 469 comments related to the research question and categorized these into the four main categories (a) ‘Preparation’, (b) ‘Process’, (c) ‘Atmosphere’, (d) ‘Value of the MT’. Interviewees highlighted the importance of a careful preparation phase, clear expectations, a clear research plan, thorough and timely feedback, mutual agreement on timelines, and a positive working atmosphere. Each of these factors could be brought in line with the three components of the EA framework: *agreement and clarity of goals, tasks, relationships & roles.* Satisfaction with the MT process was rated 8.75 ± 1.22 SD (of 10) points by supervisors, and 7.80 ± 1.61 SD points by students, while supervision quality was rated **+** 1.51 ± 0.63 SD (scale from − 2 to + 2) by supervisors, and **+** 1.26 ± 0.93 SD by students.

**Conclusion:**

We propose the EA framework as a useful guidance for students, supervisors, and the university towards conducting successful MTs in medicine. Based on the findings, we provide specific recommendations for students, supervisors, and university.

**Supplementary Information:**

The online version contains supplementary material available at 10.1186/s12909-023-04593-7.

## Background

Participating in an undergraduate research project promotes scientific competencies such as interpreting studies, critical thinking, and applying evidence-based medicine in the clinical context [1–4]. A Master Thesis (MT), or a report on a scientific project, is therefore an obligatory element of the undergraduate curriculum in countries which have adopted the Bologna framework in medicine, i.e. those that organize undergraduate medical education into a Bachelor and a Master phase [5].

From a curricular perspective, conducting a MT establishes a longitudinal one-to-one teaching relation between student and supervisor [3, 6]. For postgraduate doctoral programmes, several publications have explored this relation from various perspectives. Heyns et al. have provided advice for supervisors for guiding students in health care, [7] and recently, a literature review has addressed supervising strategies for postgraduate research in general [8]. Both papers advocate for a more person-centered approach with a focus on feedback [7, 8]. Other authors have included the students’ perspective, highlighting a mismatch between their expectations and experiences, [9] or differences between supervisors’ and students’ expectations [10]. For doctoral theses, there is consensus that a mutual understanding and an alignment of expectations is essential [11, 12] and a dialogue between the two parties should be encouraged [13].

However, this research has not yet been extended to the MT process. The student - supervisor relationship during the MT process has been addressed by only a few studies [6, 14, 15]. De Kleijn showed for the fields of social sciences, geosciences, and humanities that supervision should be carefully balanced [15]. Another paper on undergraduate engineering studies found that expectations were not well aligned between university, supervisor and student [14]. That paper found that supervisors pursued a more ‘research outcome’ orientated goal as opposed to an educational goal (to provide students with an understanding of the research process) [14]. Another study from business school addressed a lack of training for supervisors [16]. However, none of these studies on undergraduate thesis writing has offered an educational framework to outline the student - supervisor relationship [17]. In addition, conditions in medicine differ from those in these other fields as the professional identity formation in medical studies focusses on clinical competencies rather than on scientific careers [18].

Conceptualizing this mutual relationship between students and supervisors, an explicit framework such as the ‘Educational Alliance’ (EA) [19] could help both parties to better understand how to successfully shape the MT process. The EA was initially developed and extensively explored as the ‘working alliance’ in the field of psychotherapy, [20, 21] where it is accepted as a central principle across therapeutic modalities [22] with robust effects on patient outcome [23]. Recently, the concept has been adapted to the context of medical education [19, 24–26]. The EA concept builds on three components, (a) clarity and agreement on goals, (b) clarity and agreement on tasks, (c) clarity and agreement on relationship & roles. Developing a mutual agreement on all three components helps to build a strong bond in the educational relationship and may lead to improved success [19]. Given the longitudinal one-to-one relationship between supervisors and students conducting a MT, the EA model could be a useful framework for better understanding this educational process.

In this study, we sought to collect specific evidence from the field of undergraduate medical education and to test whether the findings would fit into the EA framework. We addressed two research questions, (a) Which factors facilitate, or hinder, a successful MT process? and (b) Are the findings consistent with the three components of the EA? For this purpose, we conducted semi-structured interviews independently with students and their corresponding supervisors. Based on the results, we aimed to provide guidance for students, supervisors, and the university towards a successful MT process.

## Methods

### Curricular context

Undergraduate medical curricula in Switzerland follow the Bologna structure [5] with a three-year Bachelor and a three-year Master phase. The detailed structure of the curriculum at the University of Zurich has been described elsewhere [27, 28]. The Swiss national catalogue of learning objectives [29] states for the Master thesis that “*students should be able to ‘identify and develop a research question or hypothesis, analyze, and synthesize the results, and present these as a scientific report or article”*. Students may work on the project during the whole Master phase, and they approach potential supervisors individually. Topics can be chosen freely from all types of scientific work such as clinical studies, literature reviews, basic lab science, or essays. The work may either be published as a journal article or submitted to the university as a monograph. Before starting the project, the student and supervisor must agree on a written outline of the thesis, which is submitted to the Vice Deanery of Education. The total workload accounts for 15 Credits of the European Credit Transfer System (ECTS) representing 450 working hours in total. A thesis committee decides whether to accept the thesis during the final year of studies.

### Study design

The Ethical Committee of the Canton of Zurich declared the study did not fall within the scope of the Swiss Human Research Act and therefore granted exemption (BASEC Req-2018-00345). The research was performed in accordance with the Declaration of Helsinki. All information which could have identified persons or institutions was anonymized before data analysis, and all participants gave informed written consent.

From autumn 2019 to spring 2020, we conducted semi-structured face-to-face interviews with MT students and – separately – with their individual supervisors. Hierarchical power issues between students and supervisors were minimized as the interviews were conducted separately and the MT had already been completed. No incentives for participation were provided. The participants were recruited for one part from a pool of particularly successful MTs (e.g., nominated for university awards), labelled as ‘Published MTs’ (PMTs). These MTs had been published as journal articles with the student as first or second author, listed in the ‘Science Citation Index’ (SCI) in the first or second quartile of the subject domain, with a Journal Impact Factor (JIF) [30] in 2017 above 2.0. A second category for analysis included cases where the MT thesis had only been submitted as a monograph to the university library repository (ZORA). These cases were classified as ‘Unpublished MTs’ (UMT). UMTs were randomly picked from the electronic database of the Vice Deanery of education as a convenience sample. We deliberately decided to investigate both PMT and UMT cases as we hypothesized that there could be differences in the facilitating or impeding factors between the groups.

The interviews were based on MTs submitted from 2013 to 2017, which ensured that adequate time had passed for a manuscript to be published. An interview guide was designed following Kallio et al., [31] informed by student and supervisor feedback from the preceding academic years, and subsequently refined by content experts from the university faculty. We designed two corresponding versions of the interview guide, one for students and one for supervisors. The guides were piloted with two students and two supervisors, and subsequently adjusted for minor points. This final version was used for the first 18 interviews, after which we screened the answers for redundancy and to determine if further focus was needed. We then made minor adaptations to the student questionnaire by removing two questions that were not related to individual experiences and adding one question about selecting topics and supervisors. A question not related to individual experience was also removed from the supervisor’s questionnaire. The refined interview guides are attached as Supplementary File 1.

In addition to differences between PMTs and UMTs, we compared student and supervisor responses.

### Interviews

After having obtained written permission, we started the interview by asking questions relating to overall satisfaction with the MT process and general impressions. In the main section, we posed open questions regarding the factors that facilitated and hindered the success of the MT. Additionally, we asked questions about personal aims, achievements, and the value of the MT. Students and supervisors both rated how they perceived the process of the MT on Likert-type scales from 0 (not satisfied at all) to 10 (very satisfied). Finally, students rated the perceived quality of supervision in eight Likert-type questions (from − 2, “totally disagree” to + 2, “totally agree”) while the supervisors self-rated their supervision quality using the same scale (for specific questions, see Supplementary File 2). The mean value of these eight answers was taken as a compound score for ‘perceived supervision quality’. With this, we aimed to control for supervision quality as a potential confounder.

### Data handling

The interviews were audiotaped and subsequently transcribed using the software ‘MaxQDA’ [32]. Participants did not review their transcripts (mainly, to reduce social desirability bias). All information with the potential to identify persons or institutions was anonymised.

### Qualitative content analysis

From the anonymized transcripts, qualitative content analysis was conducted according to Mayring [33]. All comments pertinent to the research question, “*Which factors and supervision strategies facilitated or hindered the progress of a Master Thesis?*”, were extracted and then sorted into main categories and further subcategories. To derive the main categories, four researchers (MB, AW, AA, JB) independently analysed 20% of the transcripts with respect to the research question. The final main categories were agreed upon after discussion by all four researchers. The comments were classified into ‘positive’, ‘negative’, or ‘neutral’. To evaluate facilitating factors, only positive and negative comments were used. Lastly, comments were compared between students and supervisors, and PMT and UMT groups.

### Sample size

We assumed that sufficient saturation of information (‘information power’ according to Malterud et al.) [34] was reached at 6–10 interview pairs per PMT and UMT group, respectively. In total, 24–40 single interviews were proposed. Given the qualitative study purpose we deliberately did not perform statistical comparison.

## Results

In summary, we conducted 40 Interviews corresponding to 20 MTs (22 PMTs and 18 UPTs). All persons invited agreed to be interviewed. 6 students were female, 14 male, and the median student age at the time of the MT submission was 25 (range 24–28). No differences were apparent between PMT and UMT or between females and males. Of the supervisors, 6 were female and 14 male. Academic ranks (and ages) were slightly higher for UMT supervisors. Further details are provided in Supplementary File 3. The median interview duration of all interviews was 27 min (range 19–42).

In the following, we outline the most important findings according to the main categories ‘Preparation’, ‘Process’, ‘Atmosphere’ and ‘Value of the MT’.

### Qualitative content analysis

From the transcripts, we extracted 469 relevant comments, 380 of which were positive and 89 negative. Table 1 gives a quantitative overview of all comments by categories and subcategories. Detailed information on subcategories, including verbatim quotes, is provided in Table 2.

**Table 1:** Categories and subcategories identified by inductive qualitative content analysis (numbers of comments per category and subcategory)

Category	Published Master Theses n = 22				Unpublished Master Theses n = 18			
subcategories	**Supervisor**		**Student**		**Supervisor**		**Student**	
	**Pos**	**Neg**	**Pos**	**Neg**	**Pos**	**Neg**	**Pos**	**Neg**
**All comments n = 469**	**131**	**21**	**83**	**21**	**101**	**16**	**65**	**31**
**Preparation n = 79**	21	6	16	0	16	4	16	0
First impression of partner	5	0	4	0	1	0	2	0
Gather information of partner	5	0	4	0	3	0	6	0
Time to think over for the student	6	0	0	0	7	0	0	0
Interests in the subject	0	0	8	0	0	0	8	0
Resources by the university	5	6	0	0	5	4	0	0
**Process n = 155**	34	13	23	11	33	8	21	12
Guidance	7	3	4	2	11	1	7	1
Clarity on Expectations	9	0	5	0	10	1	9	3
Time management	7	2	8	3	5	1	3	4
Availability of materials needed	9	3	6	6	5	3	2	4
Research skills of students	2	5	0	0	2	2	0	0
**Atmosphere n = 186**	64	2	32	8	42	2	23	13
Feedback	13	1	6	1	10	0	4	4
Reliability	7	0	8	5	5	0	5	3
Motivation Student	15	0	4	0	10	0	1	1
Motivation Supervisor	13	0	4	0	4	0	1	0
Relationship	12	0	10	1	10	0	8	2
Supervisor’s experience	4	1	0	1	3	2	4	3
**Value of the master thesis n = 49**	12	0	12	2	10	2	5	6
No sub-categories	-	-	-	-	-	-	-	-

**Table 2:** Subcategories: Detailed information and exemplary verbatim quotes

Subcategories	Driver	Quote
**First impression of partner**	A preceding interview was helpful to gain an impression if a relationship would work.	Supervisor: “*At first we met to get to know each other to see if this relationship will even work*” Student: “*I chose him based on my gut instinct. However, at first I contacted another supervisor who was really hard to communicate with*”
**Gather information of partner**	Information from fellow students and/or other sources could help to estimate the effort the working partner might be willing to invest.	Supervisor: *“[…] I asked some graduated students what they have to say about this student*” Student: “*I looked for a supervisor with good references who had already supervised several MT successfully. […]*”
**Time to think over for the student**	Supervisors made positive experiences if they provided insights of the possible topics and thereafter let the student think about the project.	Supervisor: “*First we meet and talk about the process. I always tell them to think about it for several nights and that they should send me an e-mail if they’re still interested.*”
**Interests in the subject**	Many students chose their MT according to their interests and thereby, made good experiences.	Student “*I was interested in the topic and wanted to get an exciting view in the specialization and the laboratory and their application to clinical work.*”
**Resources by the university**	Guidelines offered by the university cover the essentials but are too extensive.	Supervisor: “*The guide provided is too extensive and complicated. They should introduce a short version. Otherwise, students always come up with questions.*”
**Guidance**	Students needed basic knowledge and ideas how to get into the topic. This was best delivered in a 1:1 teaching setting, which prepared students to work autonomously.	Supervisor: “*He should be present at measurements. I told him how to look for literature and gave him some example papers.*” Students: “*The good supervision and the given support on how to look for literature were beneficial.*”
**Clarity on expectations**	It was important to clarify the estimated effort and discuss aims at the beginning. Therefore, it was helpful to first prepare a research plan together.	Supervisor: “*We had a research plan which needed to be realistic. It is important to know how the control cases are defined.*” Student: “ *… it was very helpful that it was a clearly defined topic. Together, we looked at what would be the relevant data.*”
**Time management**	It was important to plan enough time for the MT, and set deadlines. There were curricular duties that students neglected to consider.	Supervisor: “*It was hindering that the student was occupied by other study course issues. That’s where we lost some time.*” Student: “*She pushed the progress in a positive sense by setting deadlines and such. It at least helped me a lot.*”
**Availability of needed materials**	It was helpful if the data was already available, or even gathered. Also, a supportive environment was beneficial.	Supervisor: “*It was good that his data was already in the register*.” Or: “*The setup was already available […] which was really good.*” Student: “*It was advantageous that I had a place to work at the institution*.”
**Research skills of students**	Initially, most students lacked essential prerequisites. Some supervisors demanded better preparation of the students by the university.	Supervisor: “*A lot of the students come with too little knowledge about statistics, a better preparation by the university would be better*.” Another supervisor: “*It was annoying since he was lacking the basics.*”
**Feedback**	Study results and open questions should be properly discussed to lead the student in the right direction. The main intent was to let the student work independently.	Supervisor: “*We had several meetings to see if the thesis was developing in the right direction*.” Student: “*Corrections during the writing process would have been helpful.*” Or: “*Beneficial were the exact instructions what had to be done and that he took plenty of time to discuss problems*.”
**Reliability**	Supervisors and students appreci-ated if partners responded and were available within a reasonable time period.	Supervisor: “*It was very good in my opinion […] He worked very transparently and was always available.*” Student: “*The greatest advantage was that my supervisor responded very quickly to my mails*.”
**Motivation Student**	It was highly valued, if students showed engagement, an interest for the topic and tried to solve problems independently.	Supervisor: “*His enthusiasm and willingness to work were very helpful*.” Student: “*It was also very helpful that it was an interesting topic and that I could independently decide what to do.*”
**Motivation Supervisor**	It was helpful, if supervisors found satisfaction in supervising, if the project was in the supervisor’s field of interest and if supervisors showed engagement.	Supervisor: “*This project […] lay therefore in my own interests. I wanted to see the data correctly analysed and highly published*.” Student: “*I think it is very important that the supervisor shows interests in my work […] He was always in the background checking that it somehow works out well*.”
**Relationship**	A friendly and professional atmosphere where problems could be discussed without hierarchical imbalances were meaningful for the progress.	Supervisor: “*I think an advantage […] was that he was integrated in a team where we put a lot of value into a good team spirit […].*” Student: “*He didn’t look at my problems properly. I couldn’t tell him of his faults because […] we just weren’t working on eye level.*”
**Supervisor’s experience**	The result and what students might learn with the MT mainly depended on the planned project and therefore, on the supervisor’s experience.	Supervisor: “*Whether students reach their goals fully depends on the supervisor since they are the ones with the experience.*” Student: “*It depends on the project and therefore, on the supervisor if students can do all those steps.*”
**Value of the master thesis (MT)**	The MT was an important adjunct in the study course for students to learn the basics of research and to identify whether students appreciated research.	Supervisor: “*Ideally the MT is an eye opener where students realize if research is a kind of work they like*.” Student: “*It is the only opportunity for medicine students to actively see what research means*.”

#### ‘Preparation’

Both students and supervisors expressed it was highly important to select an appropriate working partner, and in this, they found an orientating interview was most valuable (student quote 1b: ‘*I chose him based on my gut instinct*’ [during the meeting]). Students stated that exchange with fellow students also helped to decide on specific supervisors. However, the main driver for students to decide on a project was a strong interest in the topic (student quote 4: ‘*I […] wanted to get an exciting view into the specialization [field]…’*). Supervisors found it important that students could think over the project for some time (supervisor quote 3: ‘*I always tell them to think about it for several nights …’*). Supervisors also commented on the resources provided online by the university (regulations, advisory handbook, best practice examples), which some regarded as redundant and confusing, and others found helpful.

With respect to the most important goals behind the MT, students and supervisors cited acquiring scientific skills and skills in scientific writing. In addition, both parties indicated an intrinsic interest in the topic was paramount. Publishing the thesis as a journal article was mentioned far more often by students and supervisors of PMT cases. In addition, students of PMTs more often stated they intended to take up a residency in the field of the MT. Further details are provided in Supplementary File 4.

#### ‘Process’

Students and supervisors found it important for students to have a clear understanding of what was expected. Both sides stated that the research plan helped to outline the expectations for content, amount of work, and deadlines (supervisor quote 7: *‘We had a research plan which needed to be realistic.’*). In addition, concise time management was found important. On this issue, more interviewees of the PMT group than of the UMT group made positive comments. According to some supervisors, students lacked the necessary basic scientific skills to autonomously conduct a MT (supervisor quote 10: ’*A lot of the students come with too little knowledge about statistics, …’* ), and some claimed that the university should have prepared students better.

#### ‘Atmosphere’

Both interviewee groups highlighted the importance of offering feedback in correcting students and offering support if needed (student quote 11b: ‘*Corrections during the writing process would have been helpful.*’). Negative comments in this respect were more frequent in the UMT group. Many comments were related to timely feedback, which helped students to maintain enthusiasm (student quote 12: *‘The biggest advantage was that my supervisor responded very quickly to my mails’*). Conversely, negative feedback was reported as lowering motivation. Two students reported negative feedback as having led to frustration and discouragement. As a further hindrance, two students of UMTs experienced a hierarchical imbalance, which made them feel unable to provide bottom-up feedback (student quote 15: *‘we just weren’t on eye level’*). In addition, students stated that a poor relationship decreased the supervisors’ opportunity to take up feedback and suggestions from students.

With respect to personal traits, both students and supervisors valued showing a high motivation for the project. Positive comments were more frequent in the PMT group. The motivational factors mentioned most by students and supervisors of both PMT and UMT cases were intrinsic interest in the topic and a desire to acquire or pass on scientific skills.

#### ‘Value of the master thesis’

Overall, interviewees attributed a high value to the MT. They expressed high to very high satisfaction with the process and the product of the thesis, with slightly higher satisfaction for supervisors and PMT cases (for details see Supplementary File 5). More PMT students and supervisors found that the value of the MT went beyond just being part of the curriculum. Most interviewees thought the MT was an important element of the curriculum that offered significant opportunities to acquire scientific skills (supervisor quote 17: ‘*Ideally the MT is an eye opener where students realize if research is a kind of work they like*.’).

### Satisfaction with the process and quality of supervision

Satisfaction with the MT process was rated with a mean of 8.75 ± 1.22 SD (of 10) points by supervisors, and 7.80 ± 1.61 points by students, while supervision quality was rated **+** 1.51 ± 0.63 SD (scale from − 2 to + 2) by supervisors, and **+** 1.26 ± 0.93 SD by students.

The quality of supervision was rated high to very high (overall mean: +1.39 on a scale from − 2 to + 2) (see Supplementary File 6). Supervisors self-rated their supervision quality slightly higher than the students did (supervision quality scale + 1.51 vs. +1.27), and in the PMT group, mean values were also higher (+ 1.46 vs. +1.32). However, the differences were small, and based on the considerations above we did not perform statistical analysis. Of note, the lowest ratings of supervisors by students were found in relation to ‘clarity of working instructions’.

## Discussion

This qualitative study explored factors that facilitate and hinder conducting a MT in medicine in the views of students and supervisors. By including both the student and the supervisor perspective, we gained a balanced picture and by conducting the interviews independently we were able to avoid potential hierarchical power issues between the parties. The interviews showed high agreement and consistency between students and supervisors. While strengthening the validity of the findings in general, the agreement between students and supervisors also points towards the importance of a mutual understanding in the sense of an educational alliance.

Using an inductive approach, we identified facilitating factors for the MT and sorted them into the main categories ‘Preparation’, ‘Process’, ‘Atmosphere’, and ‘Value of the MT’. In the following, we will discuss these findings in respect to the components of the EA (clarity and agreement on goals, tasks, relationships & roles).

### Clarity and agreement on goals

Very much in line with the EA, one of the most important success factors delineated in the main categories ‘Preparation’ and ‘Process’ was that supervisors and students aspired to reach common goals, e.g., acquiring or passing on of scientific skills. To align goals, both students and supervisors stressed that it was important to invest in a thorough preparation of the thesis. The importance of common goals is further indicated by the different motivations we found between the PMT and UMT groups. For PMTs, students and supervisors shared the aim of publishing the thesis as a journal article, which was presumably associated with higher (aligned) intrinsic motivation. Furthermore, PMT students more frequently regarded the MT as a step towards graduate training in the respective field. Achieving clarity and alignment of goals in advance definitively supports the development of an effective working alliance, and the literature outside of medical education mentions the need to address deficits in this area for better alignment between students and supervisors [14].

‘Clarity of expectations’ was another important finding from the interviews which goes in line with the EA. Linking the goals to the necessary time, engagement and resources provided a reliable framework for the MT process. Providing and maintaining this framework is an important competency that supervisors should develop (or should be trained for) [16].

Support for an effective EA could also come from the university curriculum. Our data underline the importance of carefully planning the curricular activities around the MT, including appropriate content and time points for courses in statistics, research design, and project management. In this sense, the university should complement the preparation by students and supervisors towards an effective EA.

### Clarity and agreement on tasks

Our findings reflect the EA component ‘clarity and agreement on tasks’ in a couple of subcategories under the main category ‘Process’. Students’ and supervisors’ comments on research plans, corresponding timelines, and the availability of resources highlight the significance of task clarity. In accordance, negative experiences by students mostly related to a lack of clear working instructions.

Most supervisors aimed to guide students towards increasing independence during the project, which is regarded a powerful driver for learning [35]. However, shaping this process of gradually and effectively fading out also calls for specific supervisor training [16].

### Clarity and agreement on relationships & roles

The EA component ‘clarity and agreement on relationships & roles’ is well reflected by our main category ‘Atmosphere’ including feedback quality, reliability, motivational factors, and personal relationship. All these factors were regarded as important to success by both interviewee groups. High feedback quality helped to create a reliable working atmosphere, and conversely, some of the negative comments from respondents illustrate that receiving negative or non-informative feedback hindered the development of autonomy and reduced motivation. This confirms findings from research education in STEM disciplines [35].

Also in line with the EA component of relationships & roles, students and supervisors highlighted that showing enthusiasm and being proactive (for both sides) was beneficial for progress. Similar needs for appropriate contact have been reported for educational one-to-one relationships in GP residency training, [36] or undergraduate clinical electives [27].

### Differences between groups

Overall, we found only discrete differences between PMTs and UMTs, mainly related to motivational factors such as aiming for a publication and planning graduate training in the field of the MT. Two hypotheses may explain why the differences were so remarkably low. First, students and supervisors had deliberately matched their expectations before starting the project. Therefore, PMT students with higher aspirations might have been more tolerant of frustrations during the project. Second, students and supervisors of the PMT cases had set their goals higher and were selected for interviewing based on their success, meaning that both had succeeded in their ‘aligned’ project. In this respect, the students and supervisors in UMT cases had also succeeded in their aligned (‘lower’) goals, which would explain why the differences between the two groups were relatively small. However, supervisors of UMT cases were slightly more senior with slightly higher academic ranks. It could be speculated that they had more experience in tailoring the MT process to the needs of the student. Importantly, UMTs should not be mistaken for non-success-cases, as these MTs were also successfully completed and thus likely stimulated a positive recollection of events.

Self-ratings by supervisors were slightly higher than those provided by students, which fits with the literature showing inconsistent correlations between supervisor and student ratings of teaching quality [37]. The major explanation given for this is that the constructs for supervision quality differ between students and supervisors [37].

### Implications

With this study, we identified factors and strategies facilitating the MT process. The findings imply that the EA could serve as a valuable framework to guide the process. Incorporating these findings could address deficits outlined by the existing literature on undergraduate thesis writing, such as poor alignment of expectations or a lack of supervisor training [14, 16]. The EA could also contribute to a more holistic understanding of *doctoral* student - supervisor relationships and could be a helpful framework for supervisor training [9–13].

Based on our findings, we provide practical recommendations for each of the three parties involved (students, supervisors, and the university) summarized in Table 3.

**Table 3:** Recommendations for students, supervisors, and the university

	Recommendations
**Students**	• Motivational factors have a high impact on success: -set appropriate goals prior to starting your MT -plan your MT according to your personal interests and future plans • Gather information about your supervisor in advance. • Ask for and accept feedback. Do not hesitate to offer bottom-up feedback.
**Supervisors**	• Prepare a research plan together with the student and formulate clear expectations to guide the student towards autonomy. • Highly value the power of feedback. Provide an adequate environment for feedback. Deliver thorough and timely feedback. • Set deadlines to enable an effective process. This binds both partners to a timeline and is part of the teaching environment.
**University**	• Orient students and supervisors towards using the MT as preparation for a doctoral thesis or to aim at a publication. • Design relevant preceding courses for basic scientific skills within the curriculum to achieve better timing in relation to the MT. As an alternative, a bachelor’s thesis could be introduced. • Introduce an anonymized evaluation (and feedback) tool for students to assess and support supervisors.

### Strengths and Limitations

As one strength of this study, we interviewed students and supervisors of the same MTs, thereby analyzing the MT process from two perspectives. As a second strength we compared PMTs and UMTs, contrasting potentially more successful projects to standard outcomes. For this purpose, we based our definition on publication metrics, although these metrics have been criticized for good reasons [38] and are not the only way to define success [39]. Notably, some research fields in medicine have differing publication cultures and are thus likely under-represented.

A weakness of this study may be the single center setting with limited generalizability, given that other universities might lay a different focus on research activities. As a second limitation, we did not actively search for poorly conducted or failed MTs to compare with the success cases. Further potential biases include a selection bias and positive recall bias since the experiences took place at least two years ago. Further research should address whether faculty development based on the EA framework could improve satisfaction with and success of MTs. In addition, the significance and impact of each of the components of the EA could be analysed in detail.

## Conclusions

We conducted semi-structured interviews with 20 students and 20 supervisors of MTs in undergraduate medical education to determine the facilitating and hindering factors for the MT process. The factors derived from qualitative content analysis were then related to the framework of the Educational Alliance (EA; drawing on ‘clarity and agreement on goals, tasks, and relationship & roles’). We found that the EA clearly serves as a valuable concept to address important shortcomings of MT processes. We therefore recommend the EA as a useful guide for students, supervisors, and the university in conducting successful MTs.

### Electronic supplementary material

Below is the link to the electronic supplementary material.

Supplementary Material 1

Supplementary Material 2

## Data Availability

The datasets used and/or analysed during the current study are available from the corresponding author on reasonable request, or are included in the supplementary information files of this article.

## Abbreviations

EA: Educational Alliance
ECTS: European Credit Transfer System
JIF: Journal Impact Factor
MT: Master thesis
PMT: published master thesis
SCI: Science Citation Index
UMT: unpublished master thesis
STEM: science, technology, engineering, and mathematics (disciplines)
ZORA: Zurich Open Repository and Archive
