# Supplementary material for: Does the ‘Educational Alliance’ conceptualize the student - supervisor relationship when conducting a master thesis in medicine? An interview study
Source: BMC Med Educ. 2023 Aug 28;23:611. doi: 10.1186/s12909-023-04593-7 (PMC10464293; doi:10.1186/s12909-023-04593-7)
Supplement: Supplementary file 1 — Supplementary Material 1 [file 12909_2023_4593_MOESM1_ESM.pdf]

Academic Achievement Master Thesis in Medicine (UZH)

Interview Guide Students - (including Notes) July 2018

Interviews in German (planned time approx. 30 min)

Keyquestion: Which factors were conducive/beneficial to "success" which were hindering.

Introduction

Obviously, you finished your master thesis successfully. Congratulations!

Some aspects certainly had a positive influence on the project, others were a hindrance. With this interview, we try to derive recommendations from your experiences.

To do this, I will now ask you some standardized question to which there are no false or correct answers.

For further evaluation, I will record this interview. I will later transcribe it anonymously and thereafter delete the file. Therefore, others will not be able to identify the involved people, institution, or the research project.

Do you agree to go on?

Date / Signature for consent

**Part A. Demographics & characteristics of thesis** *(may be filled in beforehand)*	
General classification of the thesis: clinical / clinical-experimental / basic science / epidemiology / humanities	Age of the student at the time of the submission (y)<br><br>Male / Female
Study design (qualitative / quantitative)	Result of the thesis (note, publication / IF, Q1-4, publication / review etc)
**Part B. Master Thesis as a format of teaching/learning and assessment**	
**Opening / Icebreaker:** How would you quite spontaneously describe your overall experience "master thesis" in a sentence or a term?	
How satisfied are you in retrospect with the overall MT process?<br>**0 1 2 3 4 5 6 7 8 9 10**<br>(„totally unsatisfied") to („fully satisfied")	
How satisfied are you in retrospect with the topic of the MT (your task)?<br>**0 1 2 3 4 5 6 7 8 9 10**<br>(„totally unsatisfied") to („fully satisfied ")	
How satisfied are you in retrospect with the supervisor?<br>**0 1 2 3 4 5 6 7 8 9 10**<br>(„totally unsatisfied") to („fully satisfied ")	
Did you have specific aims with this MT?	(Personal? / self-set learning objectives?)
What criteria did you use to select your supervisor?	Content / person?

Did you find a suitable supervisor in the first try?	If no: why – Theme / Gut feeling
Was there a clear research plan at the beginning? Were you able to keep it?	if no: Reason for delay? / Relevant delay? (Patient recruitment, study design, failed attempts)
Was the MT topic continued as a dissertation?	If no: reason: supervisor, theme, others

Part C. Personal experiences

What do you think you learned in this MT?	Possible answers:<br>*Development of a research question*<br>*Literature research*<br>*Evaluation / processing of the respective literature*<br>*Lab methods*<br>*Apply statistics*<br>*Writing a scientific paper*<br>*Time management*<br>*Frustration tolerance*<br>*Teamwork*<br>*Scientific reasoning / discourse*<br>*Scientific thinking*
Which factors facilitated the process, and which proved to be a hindrance?	beneficial<br><br>hindering
How would you describe the relationship with the supervisor?	Quantitative (very bad – neutral – very good): 0-5-10<br><br>qualitative:

	**Totally dis-agree**		**50%**		**Totally agree**
He / she ensured a clear working plan	□ **-2**	□ **-1**	□ **0**	□ **+1**	□ **+2**
He / she has adhered to the agreements and the work plan according to the needs	□ **-2**	□ **-1**	□ **0**	□ **+1**	□ **+2**
He / she was always well prepared (e.g., informed about study progress).	□ **-2**	□ **-1**	□ **0**	□ **+1**	□ **+2**
He / she always made it clear which work steps had to be done	□ **-2**	□ **-1**	□ **0**	□ **+1**	□ **+2**
He / she always made it clear why certain work steps had to be done	□ **-2**	□ **-1**	□ **0**	□ **+1**	□ **+2**
He / she always provided support when needed for certain work steps	□ **-2**	□ **-1**	□ **0**	□ **+1**	□ **+2**
He / she created a positive working atmosphere	□ **-2**	□ **-1**	□ **0**	□ **+1**	□ **+2**
He / she had a reasonable appreciation for my work	□ **-2**	□ **-1**	□ **0**	□ **+1**	□ **+2**
The intensity of care was	Far too low	Too low	Just right	Too high	Way too high

Were you involved in a team?	(0-10)	(if so, how well?)
How was the relationship to the institutional chair?	Quantitative (very unsatisfied – very satisfied): 0-10<br>(or) qualitative:	

**Part D. General assessment**	
Domain Master thesis	
In your opinion, what educational goals should a MT fulfil?	
What significance did the MT have in the context of your studies?	
Would you change anything fundamental about the MT in its current format?	
Should the MT be removed?	**Yes / No**

Domain 'philosophy of science'	
How would you define the term "scientificness"?	E.g. Keyword: principle „cause – effect" / logical argumentation (evidence) / Independence from the subject of research, socio-critical basic attitude
From your point of view, what are essential criteria of scientificness?	(Objectivity, reproducibility, originality / innovation, independency)
In your opinion, what are essential criteria of good scientific practice?	

Thank you very much for your help!
Would you like to say something important about the subject MT, something that I forgot to ask?

On behalf of the entire research team, thank you very much for your participation and input to further improve the master thesis

Academic Achievement Master Thesis in Medicine (UZH)
Interview Guide Supervisors (not "Chairs") - including Notes

July 2018

Interviews in German (planned time: approx. 30 min)

Keyquestion: Which factors were conducive/beneficial to "success" which were a hindering?

Introduction

Your master student finished his thesis successfully – congratulations!

Some aspects certainly had a positive influence on the project, others were a hindrance. With this interview, we try to derive recommendations from your experiences.

To do this, I will now ask you some standardized question to which there are no false or correct answers.

For further evaluation I will record this interview. I will later transcribe it anonymously and thereafter delete the file. Therefore, others will not be able to identify the involved people, institution, or the research project.

Do you agree to go on?

Date / signature for the consent

Part A. Demographics & characteristics of thesis *(may be filled in beforehand)*

General classification of the thesis: clinical / clinical-experimental / basic science / epidemiology / humanities	Age of the student at the time of the submission (y)<br>Male / Female
Study design (qualitative / quantitative)	Result of the thesis (note, publication / IF, Q1-4, publication / review etc)

**Data about the supervisor**	Academic degree
**Age** (30-39; 40-49; 50-59; 60-69)	Publications (bis 5; 6-15; 16-50; > 50)
Male / Female	Previously supervised master theses (n=1; 2-5; > 5)

Part B. Master Thesis as a format of teaching/learning and assessment

Opening / Icebreaker: How would you quite spontaneously describe this learning objective "master thesis" in a sentence or a term?

How satisfied are you in retrospect with the course of this thesis?

0 1 2 3 4 5 6 7 8 9 10

(„totally unsatisfied") to („fully satisfied")

How satisfied are you in retrospect with the concept or the topic of this thesis?

0 1 2 3 4 5 6 7 8 9 10

(„totally unsatisfied") to („fully satisfied")

How satisfied are you in retrospect with the student?

0 1 2 3 4 5 6 7 8 9 10

(„totally unsatisfied") to („fully satisfied")

Did you pursue any specific learning objectives with this student?<br>Didactic (e.g. scientific work, writing a thesis, promoting students interested in research?) publication	
If you consider the MT as a teaching format, how would you describe your teaching concept?	Close structural supervision vs. let the student make the experience
Do you know the CanMEDS-Model?	Yes / No
Did you have any personal goals / or for the institution with this MT?	
How did you select your student**?**	e.g., standard procedure?
Was there a clear research plan at the beginning? Were you able to keep it?	If no: relevant delay?<br>Reasons (patient recruitment, study design, failed attempts)?
Was the MT topic continued as a dissertation?	

**Part C. Personal experiences**	
In the specific case of this MT. What do you think the student has learned during the process?<br><br>Was the learning experience "successful"? (How would you define that?)	*expectations*:<br>development of a research question<br>literature research<br>evaluation / processing of the literature<br>Lab methods<br>Statistical applications<br>Writing a scientific thesis<br>Time management<br>Frustration tolerance<br>Team work<br>Scientific reasoning / discourse<br>Scientific thinking
Which factors facilitated the process, and which proved to be a hindrance?	Beneficial<br><br>Hindering
How would you describe your working relationship to this student?	Quantitative (very bad – very good): 0-10<br>Qualitative
Did the student work in a team?	If yes, how did it go?
How would you assess the commitment of this student?	Passive (just did the necessary)<br>Proactive (Inputs by student, new ideas)
How would you describe the attitude of this student towards science?	Negative – indifferent – open / positive– very positive

How would you rate your supervision style with this student?					
	**Totally dis-agree**		**50%**		**Totally agree**
I provided a clear working plan for this student	☐ **-2**	☐ **-1**	☐ **0**	☐ **+1**	☐ **+2**
I was able to meet the ***agreements and working plan*** appropriate to the needs of the thesis	☐ **-2**	☐ **-1**	☐ **0**	☐ **+1**	☐ **+2**
I was always well prepared and knew about the study progress	☐ **-2**	☐ **-1**	☐ **0**	☐ **+1**	☐ **+2**
I always knew exactly what to do next in the process	☐ **-2**	☐ **-1**	☐ **0**	☐ **+1**	☐ **+2**
I always made it clear why we had to take certain steps	☐ **-2**	☐ **-1**	☐ **0**	☐ **+1**	☐ **+2**
I always gave assistance for specific steps if the student needed help	☐ **-2**	☐ **-1**	☐ **0**	☐ **+1**	☐ **+2**
I created a positive working atmosphere	☐ **-2**	☐ **-1**	☐ **0**	☐ **+1**	☐ **+2**
I gave appropriate appreciation of the student's work	☐ **-2**	☐ **-1**	☐ **0**	☐ **+1**	☐ **+2**
The supervision intensity was	Far too low	Too low	Just right	Too high	Way too high

In your opinion, what task / role did you perform for your student?	Motivator? / Role model? / Guide?
How satisfied are you in retrospect with the assistance of the medical faculty of Zurich?	(Insufficient – very good): 0-10<br>Handbook / Ethics / Scoring system / Feedback from the deanery

**Part D. General assessment**	
Domain master thesis	
What educational objectives should the MT accomplish for each student?	Dependent from previously acquired knowledge / learning objectives for the student
Do you think the MT is essential in the curriculum of a medicine student?	How do you determine the importance?
Would you want to change anything about the MT if you could??	
Should the MT be removed?	Yes / No

Domain ‘philosophy of science’	
How would you define the term “scientificness”?	E.g. Keyword: principle „cause – effect“ / logical argumentation (evidence) / Independence from the subject of research, socio-critical basic attitude
In your opinion, are there any essential criteria for “scientificness”?	(Objectivity, reproducibility, originality / innovation, independency)
In your opinion, are there any essential criteria for good scientific practice?	

Thank you very much for your help!
Would you like to say something important about the subject MT, something that I forgot to ask?

On behalf of the entire research team, thank you very much for your participation and input to further improve the master thesis
