# Supplementary material for: Does the ‘Educational Alliance’ conceptualize the student - supervisor relationship when conducting a master thesis in medicine? An interview study
Source: BMC Med Educ. 2023 Aug 28;23:611. doi: 10.1186/s12909-023-04593-7 (PMC10464293; doi:10.1186/s12909-023-04593-7)
Supplement: Supplementary file 2 — Supplementary Material 2 [file 12909_2023_4593_MOESM2_ESM.docx]

**Supplementary Files 2-6**

**for Supplementary File 1**: see extra file

**Supplementary File 2**: Students’ rating and supervisors’ self-rating of supervision quality. Overall scores related to specific Likert-type questions.

|  | Questions | overall score | |
| --- | --- | --- | --- |
|  |  | students | supervisors |
| 1 | He / she ensured a clear working plan | 0.97 | 1.19 |
| 2 | He / she has adhered to the agreements and the work plan according to the needs | 1.19 | 1.39 |
| 3 | He / she was always well prepared (e.g., informed about study progress). | 1.07 | 1.36 |
| 4 | He / she always made it clear which work steps had to be done | 1.59 | 1.37 |
| 5 | He / she always made it clear why certain work steps had to be done | 0.96 | 1.59 |
| 6 | He / she always provided support when needed for certain work steps | 1.54 | 1.59 |
| 7 | He / she created a positive working atmosphere | 1.29 | 1.80 |
| 8 | He / she had a reasonable appreciation for my work | 1.52 | 1.85 |

**Supplementary File 3**

**Supplementary File 4**: Most frequent aims of students and supervisors

|  | Published Master Theses (n = 22) | | Unpublished Master Theses (n = 18) | |  |
| --- | --- | --- | --- | --- | --- |
|  | Supervisors | Students | Supervisors | Students | Total |
| Publication | 10 | 6 | 3 | 1 | **20** |
| Scientific writing | 10 | 5 | 5 | 2 | **22** |
| Acquire scientific skills | 10 | 3 | 7 | 3 | **23** |
| Gain knowledge of topic | 3 | 3 | 5 | 6 | **17** |
| Working independently | 5 | - | 3 | - | 8 |
| Intrinsic interests in the topic | 7 | 8 | 6 | 6 | **27** |
| Promote young academics | 6 | NA | 2 | NA | 8 |
| Just finish thesis | NA | 4 | NA | 2 | 6 |
| Stick to Deadline | NA | 3 | NA | 2 | 5 |
| Career | NA | 5 | NA | 1 | 6 |

**Supplementary File 5**: Satisfaction with different aspects of the Master Thesis (ranging from 0 ‘totally un-satisfied’ to 10 ‘fully satisfied’)

|  |  | Satisfaction with the | | |
| --- | --- | --- | --- | --- |
|  |  | Master thesis as a whole | Process of the master thesis | Topic of the master thesis |
|  |  | Likert scale score (max. 10 pts; + SD) | | |
| Published Master Theses | Supervisors (n=11) | **8.55** (0.93) | **8.55** (1.21) | **9.18** (0.75) |
|  | Students (n=11) | **7.64** (1.03) | **7.73** (1.19) | **8.36** (1.12) |
| Unpublished Master Theses | Supervisors (n=9) | **8.11** (2.15) | **9.00** (1.12) | **9.33** (1.12) |
|  | Students (n=9) | **7.11** (1.54) | **7.89** (1.76) | **7.78** (2.11) |
| Overall average |  | **7.88** (1.49) | **8.28** (1.38) | **8.68** (1.42) |

**Supplementary File 6:** Perceived supervision quality: supervisor self-rating, and rating by students, Published MT Cases, and Unpublished MT Cases (mean values of aggregated score from 8 items, ranging from -2 (‘strongly disagree’) to +2 (‘strongly agree’))
